# Key infection stages defending heat stress in high-temperature-resistant *Blumeria graminis* f. sp. *tritici* isolates

**DOI:** 10.3389/fmicb.2022.1045796

**Published:** 2022-11-11

**Authors:** Meihui Zhang, Aolin Wang, Cheng Zhang, Fei Xu, Wei Liu, Jieru Fan, Zhanhong Ma, Yilin Zhou

**Affiliations:** ^1^State Key Laboratory for Biology of Plant Diseases and Insect Pests, Institute of Plant Protection, Chinese Academy of Agricultural Sciences, Beijing, China; ^2^Department of Plant Pathology, College of Plant Protection, China Agricultural University, Beijing, China; ^3^Department of Plant Protection, College of Agriculture and Foresty Science and Technoloy, Hebei North University, Zhangjiakou, China; ^4^Institute of Plant Protection, Henan Academy of Agricultural Sciences, Key Laboratory of Integrated Pest Management on Crops in Southern Part of North China, Ministry of Agriculture and Rural Affairs of the People’s Republic of China, Zhengzhou, China

**Keywords:** *Blumeria graminis* f. sp. *tritici*, high-temperature-resistant isolate, infection stage, histological observation, expression level, heat shock protein

## Abstract

With the increase of temperature in the winter wheat-growing regions in China, the high-temperature-resistant *Blumeria graminis* f. sp. *tritici* (*Bgt*) isolates developed in the fields. To clarify the key infection stages and the roles of heat shock protein (HSP) genes of high-temperature-resistant *Bgt* isolates defending high temperature, 3 high-temperature-resistant and 3 sensitive *Bgt* isolates were selected from 55 isolates after determination of temperature sensitivity. And then they were used to investigate the infection stages and the expression levels of *HSP* genes, including *Bgthsp60*, *Bgthsp70*, *Bgthsp90,* and *Bgthsp104*, at 18°C and 25°C. The formation frequency of abnormal appressoria and inhibition rate of haustoria formation of high-temperature-resistant isolates at 25°C were lower than those of high-temperature-sensitive isolates, while major axis of microcolonies of high-temperature-resistant isolates was higher than those of high-temperature-sensitive isolates at 25°C. The results indicated that haustoria formation and hyphal expansion were the key infection stages of defense against heat stress in high-temperature-resistant isolates. Further analyses of *HSP* genes found the expression levels of *Bgthsp60* and *Bgthsp70c* were upregulated at 24 and 72 h post-inoculation in high-temperature-resistant isolates, while no significant difference was observed for *Bgthsp90* and *Bgthsp104* genes. Taken together, the basis of high-temperature-resistant *Bgt* isolates is associated with induced expression of *Bgthsp60* and *Bgthsp70c* response to heat stress in haustoria formation and hyphal expansion stages.

## Introduction

Temperature is an important abiotic factor influencing several plant pathogens as various features, such as geographical range, growth rates in population, infection stages, and ability to spread, ultimately affects the prevalence of the disease (Coakley et al., 1999; Velásquez et al., 2018). For example, severity and frequency of wheat yellow rust caused by *Puccinia striiformis* f. sp. *tritici* (*Pst*) increased in association with increased winter temperatures and lower spring temperatures in the USA (Coakley, 1979). Prevalence of *Pst* was decreased with increased average annual temperatures from 1950 to 1995 (Luck et al., 2011). Increased severity of wheat spot blotch caused by *Cochliobolus sativus* was associated with increased average night-time temperatures in South Asia (Sharma et al., 2007). Needle blight of pine caused by *Dothistroma septosporum* was moving north with increasing temperature and precipitation in Canada (Woods et al., 2005). To survive temperature increasing, organisms, including plant pathogens, have developed the ability to adapt changing environment by constantly adjusting their phenotype on biological, ecological, and evolutionary processes (Knies et al., 2006; Knies and Kingsolver, 2010). For instance, high temperature affected conidia germination of *Pst*, *P. recondita* f. sp. *tritici*, *Beauveria bassiana*, and *Leveillula taurica* (de Vallavieille-Pope et al., 1995; Guzman-Plazola et al., 2003; Devi et al., 2005), appressoria formation in *Colletotrichum gloeosporioides* (Estrada et al., 2000), germ tube elongation of *L. taurica* (Guzman-Plazola et al., 2003), and haustoria formation in *Oidium heveae, Podosphaera xanthii*, and *Golovinomyces orontii* (Trecate et al., 2019; Cao et al., 2021). In molecular level, heat shock proteins (HSPs) resisted the effects of heat stress by protecting proteins from aggregation and degradation in many fungi (Tiwari et al., 2015). *HSP* genes in fungi played important roles when subjected to heat stress, such as HSP60 in *Aspergillus fumigatus* and *A. terreus* (Raggam et al., 2011), HSP70 in *Trichoderma harzianum* (Montero-Barrientos et al., 2008), HSP90 in *Fusarium graminearum* (Bui et al., 2016), and HSP104 in *Saccharomyces cerevisiae* (Parsell et al., 1994).

Wheat powdery mildew is a regionally epidemic disease, caused by the parasitic fungus *Blumeria graminis* f. sp. *tritici* (*Bgt*), which over-summer in cool, high-altitude wheat-growing areas in China. As the mean monthly atmospheric temperature of the major wheat-growing areas in China had increased from 1970 to 2012 by a mean 0.329°C per decade (Tang et al., 2017), the minimum altitude for over-summering of *Bgt* decreased during the disease survey (Li et al., 2013), suggesting that high-temperature-resistant isolates exist in the fields. Wan’s data proved this hypothesis, temperature sensitivity distribution of *Bgt* isolates in the fields in China was abnormal (Wan et al., 2010). However, the key infection stages of defense against heat stress in high-temperature-resistant *Bgt* isolates and the role of *HSP* genes in key infection stages remain unknown.

In this study, conidia germination, appressoria formation, haustoria formation, and hyphal expansion of *Bgt* isolates with different temperature sensitivity at 18°C and 25°C were investigated. And then expression levels of *HSP* genes were analyzed in these infection stages, including *Bgthsp60*, *Bgthsp70*, *Bgthsp90,* and *Bgthsp104*. The purposes of the present study were to clarify the key infection stages of *Bgt* isolates affected by high temperature and roles of *HSP* genes in response to heat stress. These results will help us to understanding the molecular basis of high thermal resistance mechanism in *Bgt*.

## Materials and methods

### Isolates and cultivation

*Bgt*-infected wheat leaves were collected from fields in Beijing, Shaanxi, Henan, and Yunnan province/city in China. After isolation and purification as described previously (Xu et al., 2014), 55 isolates were used in this study (Table 1). These isolates were reproduced on seedlings of highly susceptible wheat cultivar ‘Jingshuang16’, which has no effective genes against Chinese *Bgt* isolates. Briefly, seeds were sown in a Φ 5 cm glass tube covered with 5 layers of gauze to prevent accidental contamination. Conidia of *Bgt* isolates were used to inoculate on seedlings at one-leaf stage and incubated in a growth chamber (Panasonic, Ehime-ken, Japan, temperature fluctuation range: ±0.5°C) with a 16-h-light/8-h-dark cycle at 18°C.

**Table 1:** Information of *Blumeria graminis* f. sp*. tritici* isolates used in this study.

Number	Name of isolate	Collection site	Latitude	Longitude	Altitude / m	Collection date
1	13–14–7-2-2	Shaanxi (12)	34°11′	107°40′	669	2012.12.26
2	13–14–7-1-1		34°11′	107°40′	669	2012.12.26
3	13–14–1-3-1		34°34′	108°03′	581	2012.12.26
4	13–14–3-1-1		34°52′	109°56′	352	2012.12.26
5	13–14–1-2		35°14′	110°13′	662	2012.12.26
6	13–14–3-3		34°52′	109°56′	352	2012.12.26
7	13–14–1-3-2		34°52′	109°56′	352	2012.12.26
8	13–14–2-6-1		34°52′	109°56′	413	2012.12.26
9	13–14–2-1		34°52′	109°56′	413	2012.12.26
10	13–14–1-1-1		35°14′	110°13′	662	2012.12.26
11	13–14–8-2-2		34°11′	104°40′	669	2012.12.26
12	13–14–9-1		35°51′	109°30′	473	2012.12.26
13	13–1–5-5-1-2	Yunnan (11)	25°12′	100°18′	1,695	2013.04.06
14	13–1–1-1		23°54′	100°05′	1,455	2013.02.25
15	13–1–1-3		23°54′	100°05′	1,455	2013.02.25
16	13–1–2-1		25°06′	102°71′	–	2013.02.25
17	13–1–4-1-1-1		25°08′	99°11′	1,658	2013.04.05
18	13–1–4-1-2-2		25°08′	99°11′	1,658	2013.04.05
19	13–1–3-1		23°54′	100°05′	1,455	2013.02.25
20	13–1–4-1-1-2		25°08′	99°11′	1,658	2013.04.05
21	13–1–2-2		25°06′	102°71′	-	2013.02.25
22	13–1–5-5-1-1		25°12′	100°18′	1,695	2013.04.06
23	13–1–4-1-3-1		25°08′	99°11′	1,658	2013.04.05
24	13–11–4-2-2-2	Henan (15)	34°21′	110°45′	678	2013.04
25	13–11–4-2-2-1		34°21′	110°45′	678	2013.04
26	13–11–3-1-2-1		34°40′	113°12′	343	2013.04
27	13–11–1-2-3		34°40′	113°12′	343	2013.04
28	13–11–2-1-1		34°21′	110°45′	678	2012.12.28
29	Z-13–11–31–1-1		34°48′	114°21′	76	2012.5.24
30	13–11–1-2-2		34°40′	113°12′	343	2013.04
31	13–11–3-1-1-1		34°41′	113°13′	213	2013.04
32	13–11–4-2-1-1		34°21′	110°45′	678	2013.04
33	13–11–4-2-1-3		34°21′	110°45′	678	2013.04
34	13–11–2-2-3		34°21′	110°45′	678	2013.04
35	13–11–1-1-3		34°40′	113°12′	343	2013.04
36	13–11–2-3-2		34°21′	110°45′	678	2013.04
37	13–11–1-1-2		34°40′	113°12′	303	2012.12.25
38	13–11–2-2-2		34°21′	110°45′	678	2013.04
39	13–10–11–1-2	Beijing (17)	39°34′	115°42′	115	2013.05.31
40	13–10–2-3-1		39°34′	115°42′	115	2013.05.31
41	13–10–11–1-1		39°34′	115°42′	115	2013.05.31
42	13–10–2-2-3		39°34′	115°42′	115	2013.05.31
43	13–10–5-3-2		39°34′	115°42′	115	2013.05.31
44	13–10–2-3-2		39°34′	115°42′	115	2013.05.31
45	13–10–5-2-2		39°34′	115°42′	115	2013.05.31
46	13–10–2-1-2		39°34′	115°42′	115	2013.05.31
47	13–10–3-1-2		39°34′	115°42′	115	2013.05.31
48	Z-13–10-1-6-1		39°34′	115°42′	115	2013.05.31
49	13–10–2-2-2		39°34′	115°42′	115	2013.05.31
50	13–10–5-3-1		39°34′	115°42′	115	2013.05.31
51	13–10–5-1-1		39°34′	115°42′	115	2013.05.31
52	13–10–3-2-3		39°34′	115°42′	115	2013.05.31
53	13–10–3-2-1		39°34′	115°42′	115	2013.05.31
54	13–10–3-2-2		39°34′	115°42′	115	2013.05.31
55	13–10–2-2-1		39°34′	115°42′	115	2013.05.31

### Temperature sensitivity test

Temperature sensitivity was tested using seedling sensitivity assays. Briefly, conidia of *Bgt* isolates were used to inoculate on wheat seedlings and incubated in growth chamber at 18°C, 22°C, 24°C, 26°C, and 28°C, respectively, with 18°C as the reference incubation temperature. For each temperature, 15 wheat seedlings (one leaf seedling stage) were inoculated. Disease severity (percentage of diseased area to total leaf area) was recorded at 10 days post inoculation (dpi). Disease inhibition rate (DIR) was calculated by (1−average disease severity in treatment temperature/average disease severity at 18°C) × 100%. The linear regression equation was constructed with culture temperature (*X*) as dependent variables and DIR (*Y*) as independent variables as follows:


Y=aX+b

ET_50_ represented the temperature at which the DIR (*Y*) reached 50%.

### The system of screening high-temperature-sensitive and resistant isolates

To screen the high-temperature-sensitive and resistant *Bgt* isolates, ET_50_ and DIR at 26°C were used as two indictors. First, *Bgt* isolates with the ET_50_ less than 24°C and more than 25°C were classified as candidate high-temperature-sensitive and candidate high-temperature-resistant isolates, respectively. Then, the candidate isolates with high DIR at 26°C were considered high-temperature-sensitive isolates, and those with low DIR at 26°C were considered high-temperature-resistant isolates.

### Reaction of *Bgt* high-temperature-sensitive and resistant isolates to high temperature at infection stages

The temperature ranging from 15°C to 22°C is considered the optimum temperature for development of *Bgt* (Last, 1963), while *Bgt* is rarely grown at 26°C on each leaf segment. Therefore, 25°C, a sublethal high temperature was used as the heat stress treatment, and 18°C was used as the reference temperature in histological observation and *HSPs* expression analyses. For histological observation assay of *Bgt* during infection stages, one-leaf seedlings were cut into 3.5 cm-long segments. Ninety-six leaf segments were floated on a water agar amended with 60 μg/ml benzimidazole in 10 × 10 cm plates. Two plates were evenly inoculated with around 40 mg of conidia (collected and quantified to 100 μl using a 1.5 ml Eppendorf tube) of a single isolate using a settling tower. Plates were incubated at 18°C and 25°C, respectively. Conidia germination, appressoria formation (normal and abnormal appressoria formation), haustoria formation, and hyphal expansion were examined at 8, 24, 48, and 72 h post-inoculation (hpi). Glass slide was used to catch spores during inoculation to estimate the inoculation density.

To test conidia germination, about 100 *Bgt* conidia on each leaf segment were randomly selected and the number of conidia with primary germ tubes was counted at 8 hpi. For testing appressoria formation, the number of normal and abnormal appressoria was recorded for about 100 germinated conidia on each leaf segment at 24 hpi. In addition, the length of appressorial germ tube (AGT; 24 hpi) was measured using CellSens Dimension software. The formation frequency of haustoria was calculated by the percentage of the number of haustoria to the number of inoculated spores on each leaf segment (80 mm^2^ areas) at 48 hpi. Inhibition rate of haustoria formation was calculated according to the following formula:


$$\begin{array}{l} \mathrm{Inhibition rate of haustoria formation}\;\left(\%\right)\\ =\left(\begin{array}{l} 1-\mathrm{formation frequencies of haustoria}\;\mathrm{at}\;25{}^{\circ}C\\ /\mathrm{formation frequencies of haustoria}\;\mathrm{at}18{}^{\circ}C \end{array}\right)\\ \;\times 100. \end{array}$$

The major axis of the microcolonies was presented by the average hyphal expansion width of 30 microcolonies on each leaf segment at 72 hpi using a microscope at 100× magnification. Disease severities of five leaf segments were measured at 10 dpi. Three independent repeats were performed for statistical analysis.

### Staining and histological observation

For histological observation assay of *Bgt* during infection stages, infected leaf segments by *Bgt* isolates were stained with Wheat germ agglutinin (WGA) conjugated to fluorescein isothiocyanate (Sigma-Aldrich, St. Louis, MO, USA) as described previously (Ayliffe et al., 2011). After decolorating in ethanol/acetic acid (1:1 v/v), infected leaf segments were cleared in saturated chloral hydrate until translucent, followed by soaking in 1 M KOH for 1 h and neutralized in 50 mM Tris–HCl (pH 7.5). These samples were stained with a 20 μg/ml solution of WGA, followed by rinsing with distilled water. Histological observation was conducted on fluorescence microscope Olympus BX61 (Olympus Corporation, Tokyo, Japan) with blue light excitation (450–480 nm).

### Identification of *hsp60*, *hsp70*, *hsp90,* and *hsp104* genes in *Bgt*

To identify *HSP* genes of *Bgt*, amino acid sequences of HSP60, HSP70, HSP90, and HSP104 of *Saccharomyces cerevisiae* and *Aspergillus nidulans* were employed as queries to search the *Bgt* genome under the NCBI accession GCA_900519115.1 (Wicker et al., 2013). All the sequences were submitted to Conserved Domains Database (CDD) to confirm the conserved domains of HSP family in *Bgt*.^1^ WoLF PSORT was used to predict protein subcellular localization.^2^

### RNA isolation and quantitative real-time PCR

To analyze gene expressions of *Bgthsp60*, *Bgthsp70* (*Bgthsp70a*, *Bgthsp70b,* and *Bgthsp70c*), *Bgthsp90*, and *Bgthsp104* (*Bgthsp104a* and *Bgthsp104b*), the detached leaf segments were inoculated with conidia and incubated at 18°C and 25°C, respectively, and then collected and snap frozen in liquid nitrogen at 0, 24, 48, and 72 hpi. Total RNA was extracted with Trizol reagent (Invitrogen, Camarillo, CA, United States). The first-strand cDNA was synthesized with FastKing One-Step RT-PCR Kit (TIANGEN, Beijing, China). Real-time PCR amplifications were conducted with TranStart Top Green qPCR SuperMix (TRANSGEN BIOTECH, Beijing, China) and performed on ABI 7500 real-time PCR system (Applied Biosystems Inc., Foster City, CA, United States). Relative expressions were calculated using the 2^-△△CT^ method (Livak and Schmittgen, 2001) with β-tubulin as reference gene and 0 hpi of the one independent repeats of 13–10–3-2-2 as reference sample. The primers used for quantitative PCR were listed in Table 2. Three independent repeats were performed for statistical analysis.

**Table 2:** Primers used in this study.

Gene name	Primer name	Primer sequence (5′-3′)
*Bgthsp60*	RT-*Bgt hsp60a*-F	CCGAAACAGTCAAGAATGTGG
	RT-*Bgt hsp60a*-R	CGCTCGTCGTGATGTCTC
*Bgthsp70a*	RT-*Bgt hsp70a*-F	CCCTTCATTACAGCAGACTCTTC
	RT-*Bgt hsp70a*-R	CATCCTTCAGTGCCTTTCG
*Bgthsp70b*	RT-*Bgt hsp70b*-F	GCTTACTTCAACGATTCGCA
	RT-*Bgt hsp70b*-R	CCTTCTTCAATGGTCAACAGG
*Bgthsp70c*	RT-*Bgt hsp70c*-F	GGTGCTGCTGTTCAGGGTG
	RT-*Bgt hsp70c*-R	CGGTGTTTCTTGGGATGAGC
*Bgthsp90*	RT-*Bgt hsp90a*-F	CCCTCTGACATCAACGCTG
	RT-*Bgt hsp90a*-R	TTGGGCACGAATAGGATTG
*Bgthsp104a*	RT-*Bgt hsp104a*-F	CAACGACTTTAGCAGAATACCG
	RT-*Bgt hsp104a*-R	CCTCGCAGGATAGACACCG
*Bgthsp104b*	RT-*Bgt hsp104b-*F	GCGACCTACAGCAATCGG
	RT-*Bgt hsp104b-*R	TGCGGCTTCTTCCTGACA
β-tubulin	β-tubulin-F	GACACTGTTGTTGAGCCATACA
	β-tubulin-R	GACATTACGGCAGACACCAA

### Data analysis

Two-way analysis of variance (ANOVA) with SAS software version 9.4 (SAS Institute Inc., Cary, NC, USA) was used to assess the difference in conidia germination frequencies, formation frequencies of appressoria and abnormal appressoria, haustoria formation frequencies, lengths of AGT, major axis of the microcolonies, disease severity, and genes expression levels between two culture temperatures or high-temperature-sensitive and resistant isolates. One-way ANOVA was used to test the effect of temperature on inhibition rate of haustoria formation.

## Results

### Assessments of high-temperature-sensitive and resistant isolates

**Figure 1:**
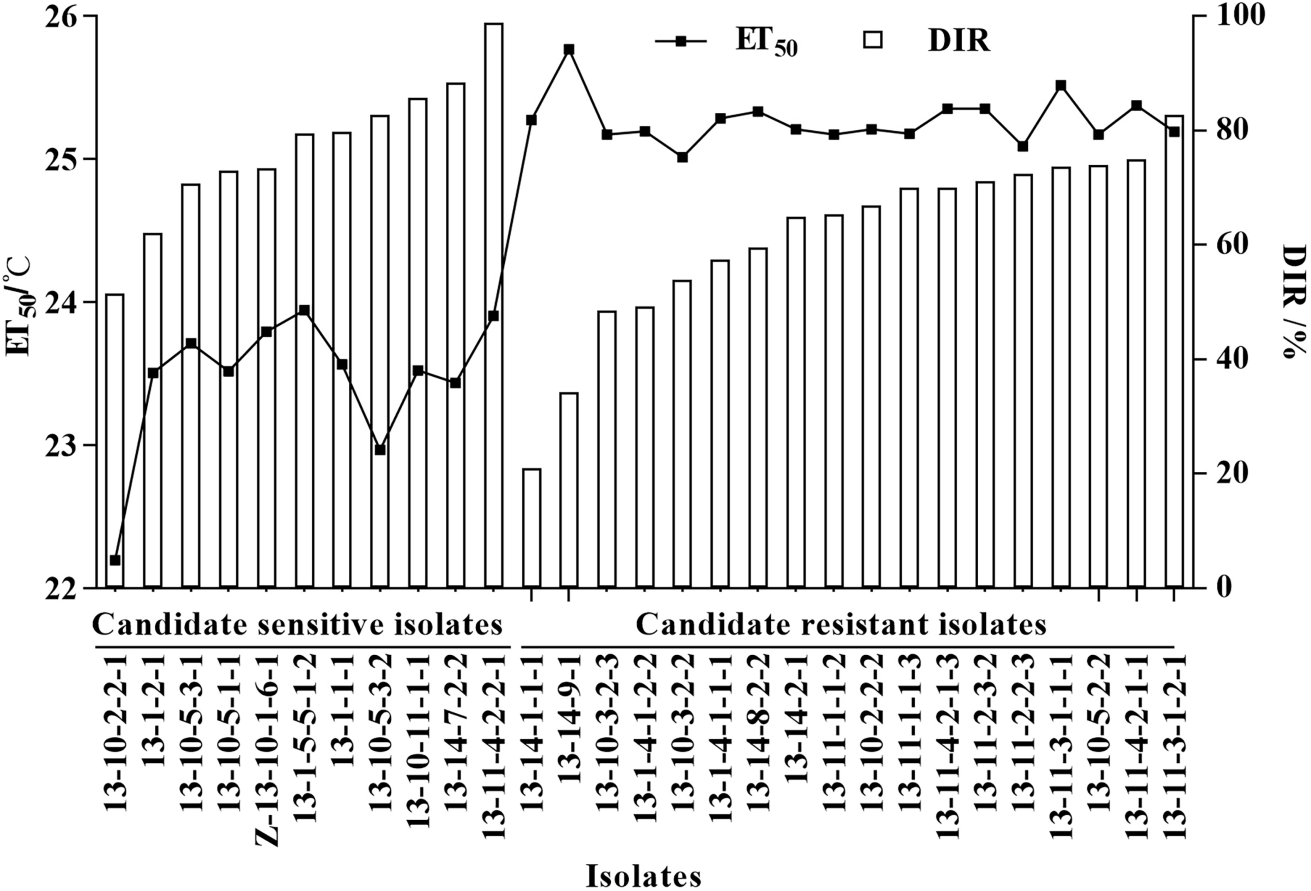
ET_50_ and disease inhibition rate (DIR) at 26°C of 11 high-temperature-sensitive and 18 high-temperature-resistant candidate isolates of *Blumeria gramini*s f. sp. *tritici*. Line chart depicted the ET_50_, and bar chart depicted the DIR at 26°C.

Temperature sensitivity tests showed that ET_50_ of the 55 *Bgt* isolates ranged from 22.20°C to 25.77°C, and mean ET_50_ was 24.57 ± 0.71°C (Table 3). Depending on the discrimination system, 11 isolates with ET_50_ less than 24°C were classified as candidates of high-temperature-sensitive isolates, and 18 isolates with ET_50_ greater than 25°C were classified as candidates of high-temperature-resistant isolates. The DIR at 26°C of those candidate high-temperature-sensitive isolates ranged from 51.54% to 98.78%, while those candidate high-temperature-resistant isolates ranged from 20.91% to 82.64% (Figure 1). Three isolates with more than 85% DIR at 26°C were selected as high-temperature-sensitive isolates, and three isolates with less than 60% DIR at 26°C were selected as high-temperature-resistant isolates (Table 3). And then, the six isolates were used in histological observation.

**Table 3:** ET_50_ and disease inhibition rate (DIR) at 26°C of 55 *B. graminis* f. sp. *tritici* isolates.

Number	Collection site	Name of isolate	ET_50_/°C	Disease inhibition rate/%	Number	Collection site	Name of isolate	ET_50_/°C	Disease inhibition rate/%
1	Yunnan	13–1–5-5-1-2	23.94	79.49	29	Shannxi	**13–14–7-2-2**	**23.44**	**88.35**
2		13–1–1-1	23.57	79.81	30		13–14–7-1-1	24.28	81.08
3		13–1–1-3	24.86	60.14	31		13–14–1-3-1	24.31	69.16
4		13–1–2-1	23.51	62.16	32		13–14–3-1-1	24.38	82.61
5		**13–1–4-1-1-1**	**25.29**	**57.41**	33		13–14–1-2	24.46	64.29
6		13–1–4-1-2-2	25.19	49.21	34		13–14–3-3	24.48	77.48
7		13–1–3-1	24.43	52.82	35		13–14–1-3-2	24.56	69.64
8		13–1–4-1-1-2	24.84	53.42	36		13–14–2-6-1	24.97	69.03
9		13–1–2-2	25.00	54.35	37		13–14–2-1	25.21	64.86
10		13–1–5-5-1-1	24.56	48.46	38		13–14–1-1-1	25.27	20.91
11		13–1–4-1-3-1	24.85	37.97	39		**13–14–8-2-2**	**25.33**	**59.62**
12	Beijing	13–10–11–1-2	24.31	95.69	40		13–14–9-1	25.77	34.26
13		13–10–2-3-1	24.37	92.80	41	Henan	13–11–4-2-2-2	23.90	98.78
14		**13–10–11–1-1**	**23.52**	**85.71**	42		**13–11–4-2-2-1**	**24.75**	**95.80**
15		13–10–2-2-3	24.22	78.54	43		13–11–3-1-2-1	25.19	82.64
16		13–10–5-3-2	22.97	82.78	44		13–11–1-2-3	24.89	82.84
17		13–10–2-3-2	24.63	74.34	45		13–11–2-1-1	24.87	79.55
18		13–10–5-2-2	25.17	73.91	46		Z-13–11–31–1-1	24.70	82.81
19		13–10–2-1-2	24.09	80.38	47		13–11–1-2-2	24.02	80.47
20		13–10–3-1-2	24.42	69.30	48		13–11–3-1-1-1	25.52	73.68
21		Z-13–10-1-6-1	23.79	73.38	49		13–11–4-2-1-1	25.38	75.00
22		13–10–2-2-2	25.21	66.96	50		13–11–4-2-1-3	25.35	70.00
23		13–10–5-3-1	23.71	70.68	51		13–11–2-2-3	25.09	72.37
24		13–10–5-1-1	23.52	72.98	52		13–11–1-1-3	25.18	70.00
25		13–10–3-2-3	25.17	48.60	53		13–11–2-3-2	25.35	71.14
26		13–10–3-2-1	24.66	51.69	54		13–11–1-1-2	25.17	65.28
27		**13–10–3-2-2**	**25.01**	**53.91**	55		13–11–2-2-2	24.57	53.55
28		13–10–2-2-1	22.20	51.54	Mean			24.57 ± 0.71	68.98 ± 15.79

High-temperature-sensitive and resistant isolates were shown in bold.

### Histological observation of infection stages of *Bgt* isolates

**Figure 2:**
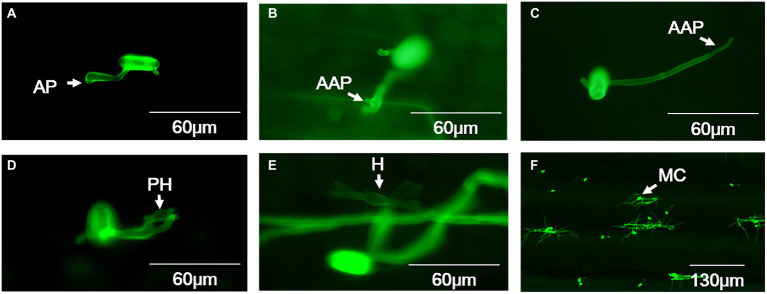
Histological observation on key infection stages of *B. graminis* f. sp. *tritici* isolates at 18°C. **(A)** Appressorium, **(B,C)** Abnormal appressoria, **(D)** Primary haustoria, **(E)** Finger-like matured haustoria, **(F)** Microcolony. AP = appressorium; AAP = abnormal appressorium; PH = primary haustorium; H = haustorium; MC = microcolony. A-E bars=60µm, F bar=130µm.

For both high-temperature-sensitive and resistant isolates incubated at 18°C, conidia germination started at 30 min post inoculation, but most of the conidia completed germination at 8 hpi, and the average germination frequency reached about 76.46% (Supplementary Table S1). About 85.22% germinated conidia had formed appressoria at 24 hpi (Figure 2A; Supplementary Table S1), after that about 12.06% appressoria were deformed (Supplementary Table S1). The abnormal appressoria were usually multi-lobed or absence of AGT hooking leading to elongation of the AGT (Figures 2B,C). Totally, around 8.46% appressoria developed to form primary haustoria (Figure 2D; Supplementary Table S1) and developed into mature haustoria with a finger-like structure at 48 hpi (Figure 2E). Then, the secondary hyphae developed from the appressoria and expanded to form microcolonies at 72 hpi (Figure 2F).

### Impact of high temperature on infection stages of high-temperature-sensitive and resistant *Bgt* isolates

**Figure 3:**
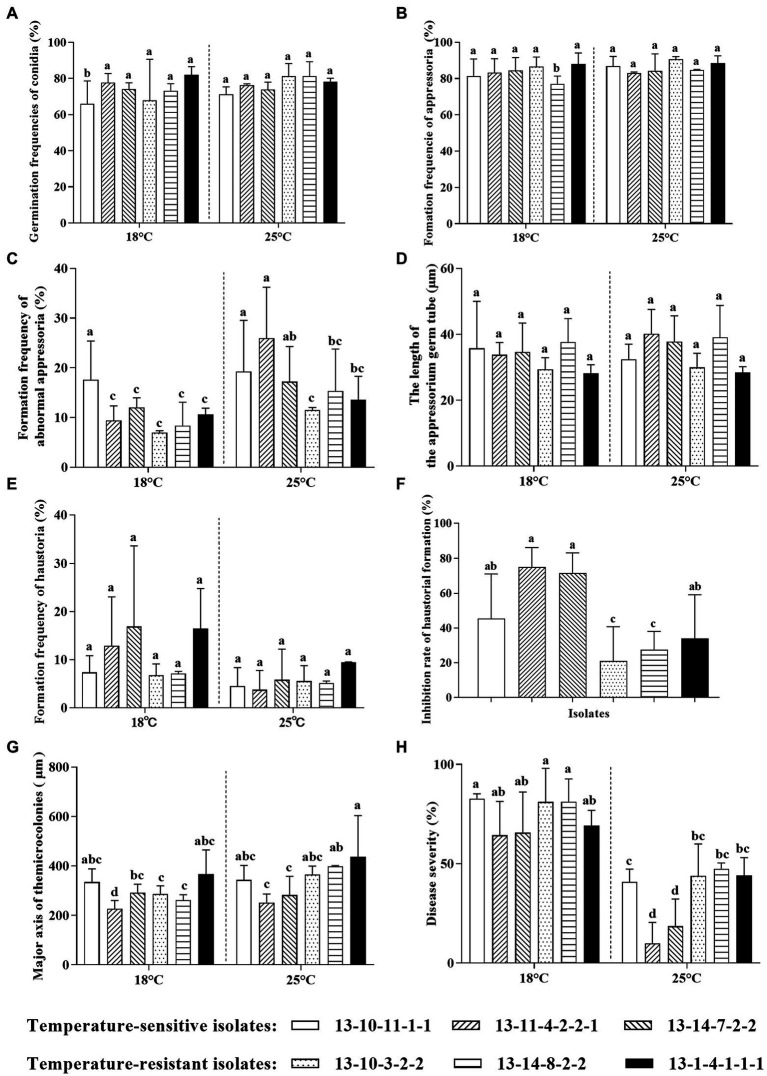
Differences in infection stages of high-temperature-sensitive and resistant isolates of *B. graminis* f. sp. *tritici* at 18°C and 25°C. **(A)** Germination frequencies of conidia; (**B)** Formation frequencies of appressoria; (**C)** Formation frequencies of abnormal appressoria; (**D)** Lengths of appressorial germ tube (AGT); (**E)** Formation frequencies of haustoria; (**F)** Inhibition rate of haustoria formation; (**G)** Major axis of microcolonies; (**H)** Disease severity. The inhibition rate of haustoria formation was analyzed by one-way analysis of variance (ANOVA), while other data were analyzed using two-way ANOVA with Duncan’s multiple range test. The error bar showed standard error of three independent repeats. The different letters above the error bars indicated significant differences (*p* ≤ 0.05).

There were no significant differences in conidia germination frequencies and appressoria formation frequencies among high-temperature-sensitive and resistant *Bgt* isolates at 25°C (Figures 3A,B). However, the formation frequencies of abnormal appressoria of high-temperature-resistant isolates were significantly lower than those of two high-temperature-sensitive isolates at 25°C (Figure 3C). In addition, there was no significant effect of high temperature on the length of AGT (Figure 3D). Furthermore, for all the high-temperature-sensitive and resistant *Bgt* isolates, about 11.30% conidia developed haustoria at 18°C, while only around 5.62% conidia developed haustoria at 25°C (Figure 3E). And the inhibition rates of haustoria formation at 25°C in high-temperature-resistant isolates (except 13–1–4-1-1-1) were significantly reduced compared with the high-temperature-sensitive isolates (Figure 3F). Moreover, major axis of microcolonies of high-temperature-resistant isolates at 25°C were higher in comparison with high-temperature-sensitive isolates (Figure 3G), which suggests that the hyphal extension of high-temperature-resistant isolates is much more easily at high temperature. Notably, disease severities of all isolates were significantly decreased at 25°C in comparison with those at 18°C; furthermore, disease severities of high-temperature-sensitive isolates were decreased more than those of high-temperature-resistant isolates at 25°C (Figure 3H).

### Identification of *Bgthsp60*, *Bgthsp70*, *Bgthsp90,* and *Bgthsp104* genes in *Bgt*

Totally, 1 *hsp60* gene, 3 homologous *hsp70* genes (*Bgthsp70a*, *Bgthsp70b,* and *Bgthsp70c*), 1 *hsp90* gene, and 2 homologous *hsp104* genes (*Bgthsp104a* and *Bgthsp104b*) were identified in the *Bgt* genome. The conserved domains of HSPs were confirmed using CDD database. There is a GroEL domain in *Bgthsp60* gene, a DnaK domain in 3 homologous *Bgthsp70* genes, a HSP83 domain in *Bgthsp90* gene, and a ClpB domain in both 2 homologous *Bgthsp104* genes. Subcellular localization prediction by WoLF PSORT showed that HSP60 was localized in mitochondria, and HSP70a, HSP70b, HSP90, HSP104a, and HSP104b were localized in cytoplasm, while HSP70c was localized in endoplasmic reticulum (ER; Table 4).

**Table 4:** Features of heat shock protein gens identified in *B. graminis* f. sp. *tritici*.

Gene name	Sequence ID	ORF length (bp)	Length (aa)	Subcellular location
*Bgthsp60*	EPQ64594	1,847	498	Mitochondria
*Bgthsp70a*	EPQ65390	1,536	511	Cytoplasm
*Bgthsp70b*	EPQ67700	1,991	648	Cytoplasm
*Bgthsp70c*	EPQ62600	2,172	578	Endoplasmic reticulum
*Bgthsp90*	EPQ66372	2,152	701	Cytoplasm
*Bgthsp104a*	EPQ63989	2,787	928	Cytoplasm
*Bgthsp104b*	EPQ65860	2,685	802	Cytoplasm

### Expression levels of *HSP* genes of *Bgt* under different temperature

**Figure 4:**
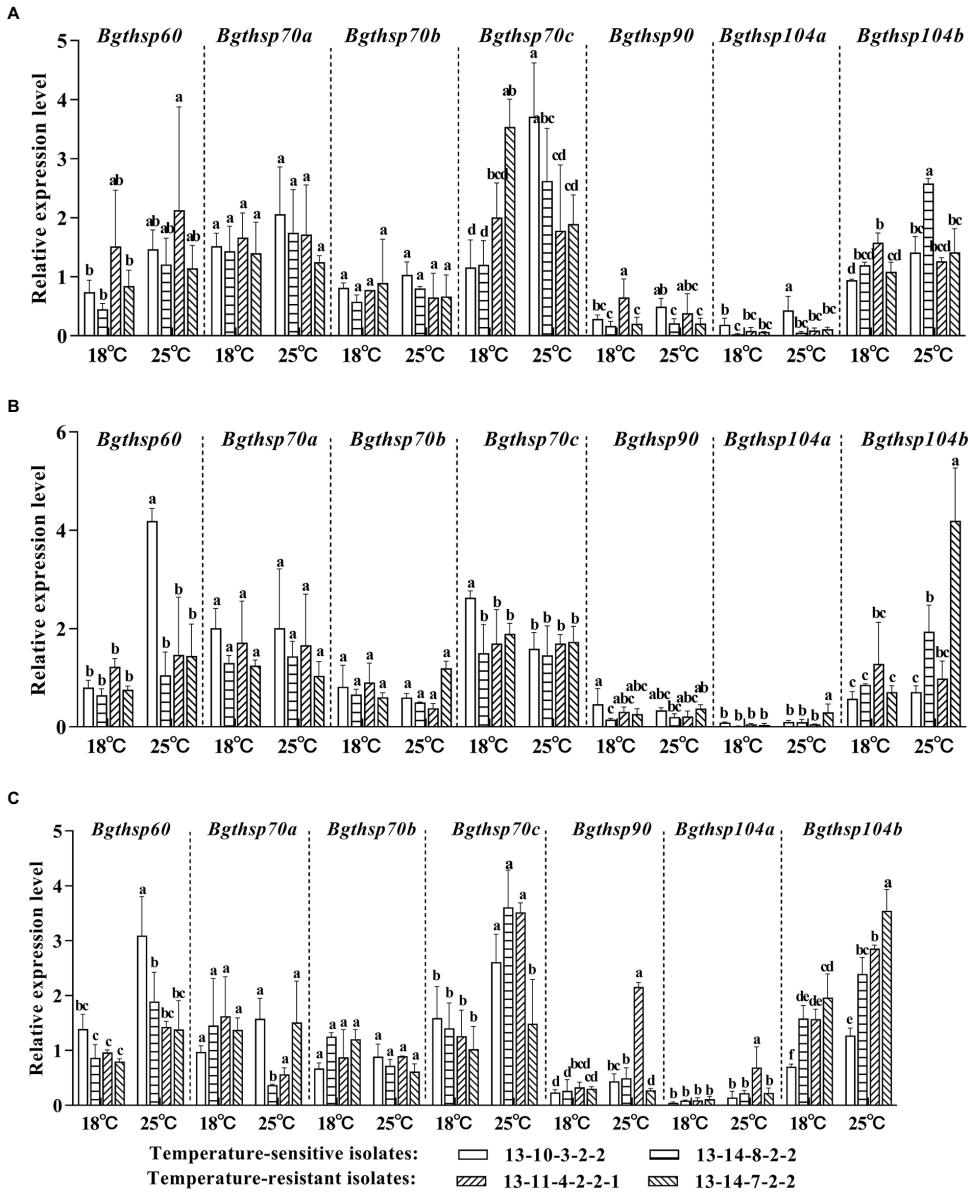
Expression levels of *Bgthsp60*, *Bgthsp70*, *Bgthsp90* and *Bgthsp104* under different temperature conditions at **(A)** 24 h post-inoculation (hpi), **(B)** 48 hpi, **(C)** 72 hpi. β-tubulin was used as reference gene and 0 hpi of the one independent repeat of 13–10–3-2-2 as reference sample. The error bar showed standard error of three independent repeats. Data were analyzed by two-way ANOVA with Duncan’s multiple range test. The different letters above the error bars indicated significant differences (*p* ≤ 0.05).

Two high-temperature-sensitive isolates, 13–14–7-2-2 and 13–11–4-2-2-1, and two high-temperature-resistant isolates, 13–10–3-2-2 and 13–14–8-2-2, were used to analyze the *HSP* genes expression levels at 24, 48, and 72 hpi at 18°C and 25°C. At 24 hpi, the expression levels of *Bgthsp60*, *Bgthsp70a, Bgthsp70c, and Bgthsp104a* in high-temperature-resistant *Bgt* isolates were increased at 25°C, while the expression levels of *Bgthsp70a, Bgthsp70c,* and *Bgthsp104a* were decreased in high-temperature-sensitive isolates at 25°C in comparison with those at 18°C (Figure 4A). At 48 hpi, expression levels of *Bgthsp60* in all *Bgt* isolates were increased at 25°C in comparison with those at 18°C (Figure 4B). At 72 hpi, expression levels of *Bgthsp60, Bgthsp70c, Bgthsp90, Bgthsp104a,* and *Bgthsp104b* in all *Bgt* isolates were increased at 25°C in comparison with those at 18°C (Figure 4C).

**Figure 5:**
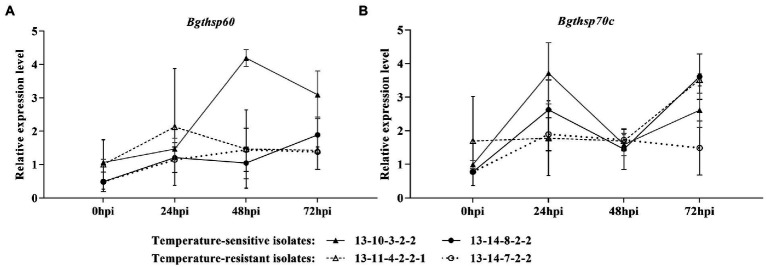
Time course of gene expression level of *Bgthsp60* **(A)** and *Bgthsp70c* **(B)** in high-temperature-sensitive and resistant isolates at 25°C. Infected leaf segments by *B. graminis* f. sp. *tritici* isolates were collected at 0, 24, 48, and 72 h post-inoculation (hpi). β-tubulin was used as reference gene and 0 hpi of the one independent repeat of 13–10–3-2-2 as reference sample. The error bar showed standard error of three independent repeats.

At 25°C, the expression levels of *Bgthsp60* were upregulation in high-temperature-resistant isolates at 48 and 72 hpi, while no difference or down-regulation in high-temperature-sensitive isolates was observed (Figure 5A). At 25°C, the expression levels of *Bgthsp70c* in high-temperature-resistant isolates were greatly increased at 24 and 72 hpi and higher than those of high-temperature-sensitive isolates at 24 hpi (Figure 5B).

## Discussion

To survive climatic warming, plant pathogens evolved to adapt changing temperature by adjusting the biology, ecology, and evolutionary processes. Although the life cycle of *Pst* will be limited by increasing temperatures, new isolates of *Pst* better adapted to high temperatures than the isolates collected before 2000 that dominated the pathogen population in south central USA (Milus et al., 2006, 2009). High-temperature-resistant *Bgt* isolates had existed in the fields in 2008 and accounted for 17.7% (20/113; Wan et al., 2010). Furthermore, the parasitic fitness of high-temperature-resistant isolates was higher than that of high-temperature-sensitive isolates under higher temperatures (Wan et al., 2012). In this research, three high-temperature-resistant and sensitive *Bgt* isolates were selected in 55 *Bgt* isolates to clarify the high parasitic fitness of high-temperature-resistant isolates at cytological and molecular levels under higher temperatures.

### Haustoria formation is the key infection stage defending heat stress in high-temperature-resistant isolates

Temperature is an important abiotic factor having a critical influence, positive, or negative, on infection stages of plant pathogens, such as conidia germination, appressoria formation, germ tube elongation, and haustoria formation in *Pst*, *Puccinia recondita* f. sp. *tritici*, *Colletotrichum acutatum*, *Leveillula taurica, Oidium neolycopersici*, *O*. *heveae, Podosphaera xanthii*, and *Golovinomyces orontii* (Guzman-Plazola et al., 2003; Leandro et al., 2003; Mieslerová and Lebeda, 2010; Trecate et al., 2019; Cao et al., 2021). This research showed that conidia germination of two types isolates was not affected at 25°C, which indicates that 25°C was not unfavorable temperature for conidia germination of *Bgt*. This is consistent with previous reports that temperatures between 15°C and 25°C were favorable for conidia germination of *B. graminis* f. sp. *hordei* on plain agar substrate (Yarwood et al., 1954). In addition, the optimum temperatures of appressorial formation of *C*. *acutatum* ranged from 17.6°C to 26.5°C, while this formation was inhibited until 30°C (Leandro et al., 2003). In this research, there were no significant differences in the formation frequencies of appressoria in two types isolates between 18°C and 25°C. This indicates that 25°C is favorable temperature as 18°C for appressorial formation of *Bgt*. These results also suggest that 25°C, a sublethal high temperature, has little effect on the precede penetration of *Bgt*.

Although the formation frequency of abnormal appressoria was increased at 25°C in comparison with at 18°C for high-temperature-sensitive and resistant isolates, the abnormal appressoria formation of high-temperature-resistant isolates was lower than that of high-temperature-sensitive isolates at 25°C, indicating that the high-temperature-resistant isolates were better adapted to high temperature. Previous research showed that the abnormal appressoria were caused by the failure of appressoria penetrating host cells (Nonomura et al., 2010), and finally resulted in the failure of haustoria formation. Although high temperature has little effect on the quantity of appressoria formation, it significantly increased abnormal morphology of appressoria, such as absence of AGT hooking and multi-lobed appressoria, followed by dramatically decreased haustoria formation frequency. A previous report also found that temperature significantly affected haustoria formation of *O. heveae* (Cao et al., 2021). In addition, the inhibition rate of haustoria formation in high-temperature-resistant *Bgt* isolates was lower than the high-temperature-sensitive isolates, suggesting that the haustoria formation of high-temperature-sensitive isolates was more severely inhibited by 25°C than high-temperature-resistant isolates. Moreover, the hyphal extension of high-temperature-resistant isolates was much more easily at 25°C than that of high-temperature-sensitive isolates. These results suggest that haustoria formation and hyphal expansion are key infection stages for high-temperature-resistant isolates defending heat stress.

### *Bgthsp60* and *Bgthsp70c* play important roles on defending heat stress in high-temperature-resistant isolates

In this study, 7 HSPs were identified in *Bgt*, of which *Bgthsp60* and *Bgthsp70c* in high-temperature-resistant isolates played important roles at 25°C in the stage of haustoria formation and hyphal expansion.

*Bgthsp60* was localized in mitochondria and significantly upregulated only in high-temperature-resistant *Bgt* isolates at 25°C compared with 18°C in the stage of hyphal expansion (Figure 4C). This suggests that upregulation of *Bgthsp60* was associated with hyphal expansion to defend heat stress in high-temperature-resistant *Bgt* isolates. Similar results were also obtained in *Aspergillus fumigatus* and *A. terreus*, the expression levels were upregulation 5.9-fold and 6.7-fold at high temperature of 40°C compared with that at regular temperature of 25°C (Raggam et al., 2011). HSP60 was required for maintaining mitochondrial protein homeostasis together with the co-chaperonin Hsp10 by preventing protein aggregation, and mediating folding and refolding under heat stress (Martin et al., 1992; Caruso Bavisotto et al., 2020). In addition, heat stress usually increased the number of interaction proteins with HSP60, which were related to metabolism demanded to proliferate in *B. graminis* (Both et al., 2005), including amino acid and protein metabolism and carbohydrate metabolism (Guimarães et al., 2011). These results suggest that *Bgthsp60* were induced by high temperature, and play a part in the process of defending high temperatures, but it remains to verify how HSP60 work in high-temperature-resistant *Bgt* isolates.

*Bgthsp70c* was identified as an ER lumenal HSP70, and upregulated in all isolates at 25°C compared with 18°C in the stage of haustoria formation and hyphal expansion. Especially, in the stage of haustoria formation, the levels of *Bgthsp70c* expression of high-temperature-resistant isolates were more than that of high-temperature-sensitive isolates at 25°C (Figure 5B). The result shows that *Bgthsp70c* of high-temperature-resistant isolates could contribute to tolerance to heat stress in the stage of haustoria formation. ER HSP70 proteins as molecular chaperones played key roles in protein transport into the ER and proper protein folding in the ER lumen, such as Kar2p and Lhs1p in fungi (Brodsky et al., 1995; Craven et al., 1996). In addition, Lhs1 was necessary for proper growth, conidiation, and pathogenicity in fungi, such as *Magnaporthe oryzae*, *Beauveria bassiana*, and *Fusarium pseudograminearum* (Yi et al., 2009; Chen et al., 2019; Wang et al., 2020). Particularly, Lhs1 properly processed of secreted proteins, including effectors, was requisite for successful disease development in *F. pseudograminearum* and *M. oryzae* (Yi et al., 2009; Chen et al., 2019). Binding protein (Bip) is also a member of the ER HSP70 family (Denecke et al., 1991). When plants were subjected to heat stress, *BiP* genes were upregulated *via* the unfolded protein response pathway, in *Arabidopsis* (Deng et al., 2011), and pepper (*Capsicum annuum L.*; Wang et al., 2017). The silencing of *CaBiP1* decreased the tolerance of pepper to heat stress. Conversely, overexpression of *CaBiP1* increased the tolerance of *Arabidopsis* (Wang et al., 2017). Therefore, it is supposed that overexpressed *Bgthsp70c* reduced the number of misfolded proteins in the stage of haustoria formation in high-temperature-resistant isolates at high temperature.

HSP90 in *F. graminearum* and HSP104 in *Saccharomyces cerevisiae* were thermotolerance factors. Deletion of *hsp90* stopped growing after heat shock (48°C for 30 min), whereas the wild-type isolates showed slightly delayed growth in *F. graminearum* (Bui et al., 2016). The ability of *S. cerevisiae* to withstand high temperatures was reduced when *hsp104* was knockout (Parsell et al., 1994). However, the expression levels of *hsp90*, *hsp104a,* and *hsp104b* showed no difference in high-temperature-resistant *Bgt* isolates at 25°C compared with that at 18°C, indicating that these genes could not be thermotolerance factors in *Bgt*.

In this research, it is confirmed that haustoria formation and hyphal expansion were key infection stages for high-temperature-resistant isolates to defend heat stress. In addition, upregulation of *Bgthsp60* and *Bgthsp70c* is associated with heat stress in high-temperature-resistant isolates in these stages. It is supposed that *Bgthsp60* and *Bgthsp70c* of high-temperature-resistant *Bgt* isolates could play a role in the process of defending high temperatures. However, the high thermal resistance mechanism of *Bgt* in haustoria formation and hyphae needs a deeper understanding.

## Data availability statement

The datasets presented in this study can be found in online repositories. The names of the repository/repositories and accession number(s) can be found in the article/Supplementary material.

## Author contributions

MZ, WL, and JF contributed to conception and design of the experiments. MZ and CZ performed the experiments. MZ, AW, WL, FX, ZM, and YZ analyzed the data. MZ wrote the manuscript. All authors contributed to the article and approved the submitted version.

## Funding

This research was financially supported by the National Natural Science Foundation of China (31972226).

## Conflict of interest

The authors declare that the research was conducted in the absence of any commercial or financial relationships that could be construed as a potential conflict of interest.

## Publisher’s note

All claims expressed in this article are solely those of the authors and do not necessarily represent those of their affiliated organizations, or those of the publisher, the editors and the reviewers. Any product that may be evaluated in this article, or claim that may be made by its manufacturer, is not guaranteed or endorsed by the publisher.

## Footnotes

^1^ https://www.ncbi.nlm.nih.gov/cdd

^2^ https://wolfpsort.hgc.jp/
